# The molecular effect of a polymorphic microRNA binding site of Wolfram syndrome 1 gene in dogs

**DOI:** 10.1186/s12863-020-00879-7

**Published:** 2020-07-28

**Authors:** Dora Koller, Eniko Kubinyi, Zsuzsanna Elek, Helga Nemeth, Adam Miklosi, Maria Sasvari-Szekely, Zsolt Ronai

**Affiliations:** 1 Comparative Ethology Research Group, MTA-ELTE, Budapest, Hungary; 2 Department of Medical Chemistry, Molecular Biology and Pathobiochemistry, Semmelweis University, Budapest, Hungary; 3 Department of Ethology, ELTE Eötvös Loránd University, Budapest, Hungary

**Keywords:** *WFS1*, Polymorphism, miRNA, Dog, Behavior

## Abstract

**Background:**

Although the molecular function of wolframin remains unclear, the lack of this protein is known to cause stress in the endoplasmic reticulum. Some variants in the Wolfram Syndrome 1 gene (*WFS1*) were associated with various neuropsychiatric disorders in humans, such as aggressiveness, impulsivity and anxiety.

**Results:**

Here we present an in silico study predicting a single nucleotide polymorphism (rs852850348) in the canine *WFS1* gene which was verified by direct sequencing and was genotyped by a PCR-based technique. We found that the rs852850348 polymorphism is located in a putative microRNA (cfa-miR-8834a and cfa-miR-1838) binding site. Therefore, the molecular effect of allelic variants was studied in a luciferase reporter system that allowed assessing gene expression. We demonstrated that the variant reduced the activity of the reporter protein expression in an allele-specific manner. Additionally, we performed a behavioral experiment and investigated the association with this locus to different performance in this test. Association was found between food possessivity and the studied *WFS*1 gene polymorphism in the Border collie breed.

**Conclusions:**

Based on our findings, the rs852850348 locus might contribute to the genetic risk of possessivity behavior of dogs in at least one breed and might influence the regulation of wolframin expression.

## Background

Investigation of the genetic background of behavior is in the center of interest for more than two decades. Psychogenetic studies of human personality began in 1996 when associations were found between a repeat polymorphism in exon III of the dopamine D4 receptor gene (DRD4) and novelty seeking [1]. Dogs are increasingly applicable models in human psychogenetic studies, not only because they live together with humans in similar social environment, but they also share various behavioral disorders, such as over-aggression, attention deficit hyperactivity disorder, anxiety and obsessive-compulsive disorder [2].

Compared to human polymorphism data, very few canine polymorphisms can be found in databases. Even though some databases exist which contain polymorphisms in canine genes, usually predicted by in silico methods, they do not have sufficient subject number or do not consider several breeds [3]. Therefore, it is of importance to confirm the existence of predicted polymorphisms by targeted sequencing. Moreover, despite of the rapid progression of genomic approaches, the genetic background of canine behavior is far from completely explored yet [4]. Several phenotypes were investigated such as dog-directed aggression, owner-directed aggression, nonsocial fear, separation anxiety and touch sensitivity [5–8]. Furthermore, associations between polymorphisms in the dopamine receptor D4 gene (*DRD4*) and the tyrosine hydroxylase gene (*TH*) and impulsivity, activity, inattention of German shepherd dogs and Siberian husky dogs; and oxytocin receptor (*OXTR*) and mu (μ) opioid receptor (*OPRM1*) genes and greeting behavior and human directed social behavior in German shepherd dogs and border collies were published by our group previously [9–12]. However, the previously mentioned studies did not investigate the molecular effects of carrying different alleles.

Several common Wolfram Syndrome 1 gene (*WFS1*) alleles were shown to associate with various neuropsychiatric disorders [13], such as bipolar depression [14], high risk to attempt suicide and aggression, or with behavioral traits, such as anxiety, impulsivity [15], and impulsive verbal and physical aggression [16] among humans. This gene encodes an endoplasmic reticulum membrane protein (wolframin), present mainly in neurons and pancreatic β-cells. The function of this protein is not completely understood yet [17], however, it is well known that its malfunction causes an increased stress in the endoplasmic reticulum, leading to the destruction of cell cycle and calcium homeostasis [18]. Rare mutations in the human *WFS1* gene, located on human chromosome 4, are responsible for the development of Wolfram syndrome, which is considered monogenic [19]. This disease is a clinical condition with the presence of diabetes insipidus, diabetes mellitus, optic atrophy and deafness [20].

Wolfram syndrome is considered a monogenic disorder, however, behavioral traits associated with polymorphisms in the *WFS1* gene are more complex [21]. To understand this complexity, the functional role of these polymorphisms should be investigated for which microRNAs (miRNAs) are suitable candidates. MiRNAs are a family of small, non-coding RNAs that play important regulatory roles in many physiological and disease processes [22]. Although 355 miRNAs are predicted in silico having a putative binding site in the 3’UTR region of the human *WFS1* gene (http://zmf.umm.uni-heidelberg.de/apps/zmf/mirwalk/), only a few of them have been confirmed by molecular methods [23]. To understand the role of miRNAs in different physiological processes, it is important to utilize experimental approaches that allow for the detection of miRNA-induced alterations in mRNA expression. Firefly luciferase is commonly used as a reporter to assess the transcriptional activity of target genes in intact cells. After its introduction to the market, the luciferase reporter gene assay was used to examine the regulation of transcriptional activities by promoters and transcription factors [24]. Currently it is also applied for testing the effect of miRNA-mediated, post-transcriptional regulation in target genes by composing a luciferase gene construct containing the predicted miRNA targeting sequence from the target gene (often located in the 3′UTR) [25].

The aim of our study was to investigate if any polymorphism in the *WFS1* gene regulates wolframin expression. Particularly, we aimed to demonstrate if there is any locus which is a functional polymorphism. Additionally, we aimed to associate this genetic marker to a behavioral trait in dogs to determine its possible role in the molecular genetics of dog behavior. According to our best knowledge, *WFS1* polymorphisms and their role have not been studied in dogs yet. Moreover, this is the first report describing the functional effect of a miRNA binding site polymorphism in *WFS1* gene in dogs.

## Methods

### Samples

In total, 274 animals were included in the study (German shepherd dogs, *n* = 104; border collies, *n* = 83; Eurasian wolves (*Canis lupus*), *n* = 34; Labrador retrievers, *n* = 23; beagles, *n* = 19; golden retrievers, *n* = 11). Buccal samples were collected from the animals as described in [26]. Briefly: the inner surface of the cheek was rubbed with a cotton swab for about 10 s, and the procedure was repeated with a second swab. DNA purification was carried out as desribed earlier for human samples [27]. Typical DNA concentration of the dogs’ genomic DNA samples isolated from buccal swabs was around 20 ng/μl.

### Sequencing

The dog *WFS1* gene sequence was obtained from GenBank (http://www.ncbi.nlm.nih.gov/) and Ensembl (http://www.ensembl.org/) databases, accession numbers were as follows: NC_006595.3 and ENSCAFG00000001781, respectively. Five sets of PCR primers were constructed by the NCBI/Primer-Blast tool (http://www.ncbi.nlm.nih.gov/tools/primer-blast/). The Qiagen Hot-StarTaq polymerase kit (Qiagen, Hilden, Germany) was used for PCR amplification. The reaction mixture included 1 μM of each primer (Table 1), approximately 5 ng of DNA template, 200 μM dNTP, 0.025 U HotStarTaq DNA polymerase, 1× buffer, and 1× Q-solution supplied together with the enzyme. The PCR cycle consisted of an initial denaturation at 95 °C for 15 min, 40 cycles of 1-min denaturation at 95 °C, 1-min annealing at different temperatures shown in Table 1, a 1-min extension at 72 °C, and a 10-min final extension at 72 °C. The total volume of the reactions was 20 μl. The obtained PCR products were cleaned with a Wizard SV Gel and PCR Clean-Up System (Promega A9282 Madison, Wisconsin, USA) and sequenced with Sanger method (Microsynth AG, Balgach, Switzerland) in both forward and reverse directions with the same PCR primers used for amplification. SNPs were identified by aligning and comparing the sequence data with the Clustal Omega tool (http://www.ebi.ac.uk/Tools/msa/clustalo).

**Table 1:** Sequencing primers and annealing temperatures used for PCR amplification of dog WFS1 gene regions

Sequencing primers	Forward primer	Reverse primer	Product lenght (base pairs)	T_**A**_ (°C)
Primer pair 1	CACCCCTGCCACATCCGCAA	CGGCCACGTCGACTCCCAAC	525	59
Primer pair 2	GTCCTGCAGCCCCGGCAATG	GCGACCAGAGGTGTCCGCAG	531	59
Primer pair 3	GGCCGCGGAGGAGCTATGC	CCACGTCGACTCCCAACGCT	562	60
Primer pair 4	ACGGCCGCGGAGGAGCTATG	GTTCCTGTGGTGCTGGTGCCC	537	60
Primer pair 5	CGGAGGAGCTATGCCGCCTGA	GCTGCAGGCAGTTCCTGTGGT	540	60

### Genotyping

The rs852850348 SNP in the dog *WFS1* 3′UTR was genotyped as follows: PCR amplification was performed with the following primers: 5′ GTCCTGCAGCCCCGGCAATG 3′ and 5′ GCGACCAGAGGTGTCCGCAG 3′. The following thermocycle was used: 95 °C for 15 min, then 40 cycles of 94 °C for 1 min, annealing at 58 °C for 30 s and 72 °C for 1 min. The final step was 72 °C for 10 min. Total reaction volume was 10 μl. PCR products were incubated for 3–6 h at 37 °C in a restriction enzyme mixture consisting of 0.5 U/μl *Pvu*II restriction enzyme (NEB), 1× BSA and 1× NEBuffer 3.1 in a total reaction volume of 20 μl. The digested PCR products were analyzed by conventional submarine agarose gel electrophoresis (Biocenter, Szeged, Hungary), using 1.5% agarose mixed with 2% MetaPhor® Agarose gel (Lonza Walkersville, Inc., USA) and visualized by ethidium bromide staining.

### Assessment of *WFS1* expression

#### Plasmid construction

A 524 base-pair long segment of the 3′ untranslated region (3’UTR) of the *WFS1* gene was amplified by PCR using a DNA-sample of a dog with known (homozygous AA) genotype for the previously identified SNP. We used the following primers: 5′ CGT TCC GAG CTC CCG TGT GAG CCC GTC C 3′ (the *Sac*I restriction enzyme site is underlined) forward and 5′ AAA TAA ACG CGT GAC CAG CAG TAG GTT TCG TGA 3′ (the *Mlu*I restriction enzyme site is underlined) reverse primers. The PCR products were digested with *Sac*I and *Mlu*I and were cloned downstream of the firefly luciferase gene in a pMIR-Report plasmid (pMIR-REPORT miRNA Expression Reporter Vector System; ABI) by T4 DNA ligase (New England BioLabs, Ipswich, Massachusetts, USA) using standard protocols. The construct with the G allele was generated by Quick Change Lightning Site-Directed Mutagenesis Kit (Agilent Technologies, Santa Clara, USA). A control construct was used containing a different insert with the same length lacking any sequence complementary to miR-8834a and miR-1838. All constructs were confirmed by Sanger sequencing.

#### miRNA binding assay

An in silico study was performed to identify miRNA binding sites in the dog *WFS1* gene. Canine mature miRNA sequences were downloaded from the miRBase database (http://www.mirbase.org/). Based on the sequence of the *WFS1* 3′ UTR, two miRNAs (cfa-miR-8834a and cfa-miR-1838) were suggested to have a putative interaction with the mRNA in close proximity to the rs852850348 locus. The Human Embryonic Kidney cell line (HEK293T) was cultured in DMEM medium (Gibco Invitrogen, Carlsbad, California, USA), supplemented with 10% bovine fetal serum (Lonza Walkersville, Inc., USA). The cells were maintained at 37 °C in a 5% CO_2_ atmosphere. For miRNA assays the cells were seeded into 24-well plates and were incubated for 24 h before transfection. Subsequently, 0.05 μg of the luciferase reporter construct were cotransfected with 0.2 μg β-galactosidase plasmid (Ambion, Kaufungen, Germany) and 5 pmol of cfa-miR1838 and cfa-miR8834a (Thermo Fisher Scientific, Waltham, Massachusetts, USA) using Lipofectamine 2000 (Invitrogen, Carlsbad, California, USA) according to the manufacturer’s protocol. Cells were collected 48 h after transfection, washed with PBS, then extracted by three consecutive freeze–thaw cycles and subsequent centrifugation. Supernatants were used for enzyme activity measurements. Luciferase activity was measured by adding 60 μl Luciferin reagent (Promega Corporation, Madison, Wisconsin USA); 0.16 mg/ml Luciferin K, 20 nM Tricine, 2.6 nM MgSO4, 0.1 nM Na2EDTA, 33.3 nM DTT, 0.27 nM Li3CoA and 0.53 nM Na2ATP) to 12 μl of each cell extract. Luminescence was measured using a Varioskan multi-well plate reader (Thermo Fisher Scientific, Waltham, Massachusetts, USA). Values for luciferase activity were normalized to β-galactosidase activity (measured by standard protocol using the same Varioskan plate reader in photometry mode). Each experiment was independently repeated three times, and each sample was studied in triplicate.

### Phenotypic measurement

**Fig. 1:**
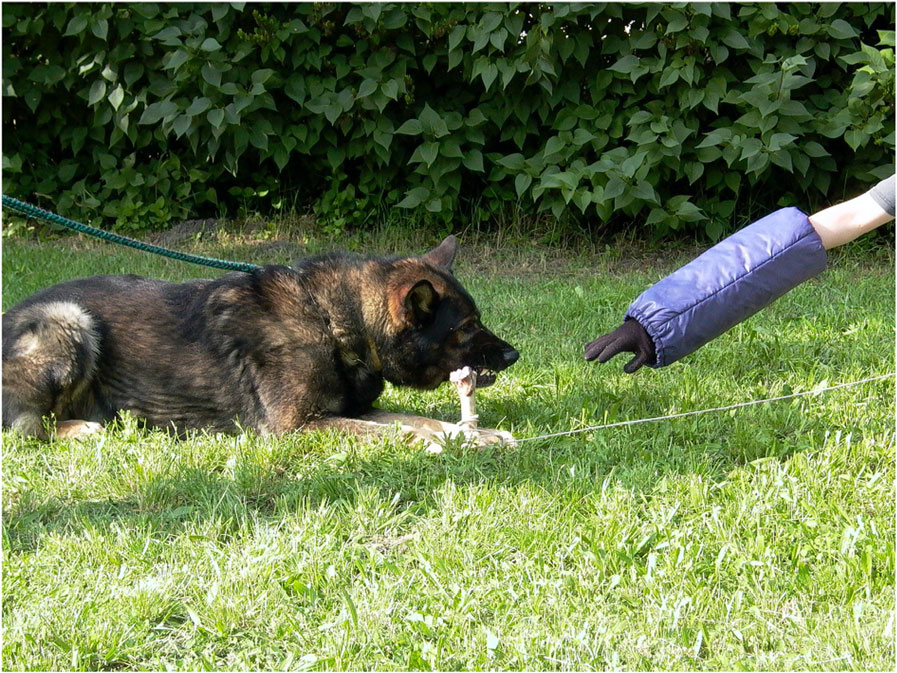
Bone take away test. Photo: courtesy of Judit Vas

Only breeds with at least 60 individuals have been included in the analysis, i.e. border collies and German shepherd dogs. The number of border collies with complete genotype, age and sex data was 64 (mean age = 4, age range: 1–9 years, male/female = 28/36); in case of German shepherd dogs the respective sample size was 72 (mean age = 3, age range: 1–8 years, male/female = 40/32). The studied dogs were individually tested in an undisturbed park in a bone take away test [28]. In the test a bone (cooked humerus of a swine) was given to the dog by the owner. When the dog was chewing the bone, the experimenter, wearing an artificial hand, approached the dog, touched the back and head of the dog, put the hand on the bone then pulled away the bone with a string attached to it. We measured at which phase the experimenter could take away the bone from the dog (‘Bone possessivity score’): when the artificial hand: was on the back of the dog (score 0); was on the bone (score 1); moved the bone (score 2); pulled away the bone from the dog (score 3). The dog was on a leash in this test, tethered to a tree in order to avoid an attack on the experimenter (Fig*.* 1).

### Statistical analyses

The Hardy–Weinberg equilibrium (HWE) for genotype distributions was assessed by χ^2^-test. No significant difference (*p* > 0.05) could be observed between the measured and expected genotype frequencies in each groups separately for the investigated SNP.

The comparison of genotype frequencies among the 5 dogs breeds and the Eurasian wolves was performed by χ^2^-tests. ANOVA of the luciferase assays was performed using SPSS software (version 17.0). *P* values lower or equal than 0.05 were considered significant. Association between *WFS1* rs852850348 and behavior was assessed by Generalized Linear Model (ordinal logistic). Sex (male, female), genotype (AA, AG, GG) were used as factors and age as covariate in the model. We analyzed the inter-rater reliability and test-retest reliability of the behavior in case of 14 dogs (independent sample) using two-way random intraclass correlation, looking for absolute agreement between average measures.

## Results

### In silico determination of rs852850348, cfa-miR-1838 and cfa-miR-8834a

A bibliographic review was carried out looking for candidates. Two DNA and 1 miRNA databases were used. Altogether, 11 SNP candidates were considered for analysis in the 3’UTR region of the dog *WFS1* gene. Amongst them, 1 resulted convenient – rs852850348 – with the highest minor allele frequency of 0.35 observed in any population. Additionally, based on miRBase database, it was a putative miRNA binding site of two miRNAs, cfa-miR-1838 and cfa-miR-8834a.

### Sequencing

**Fig. 2:**
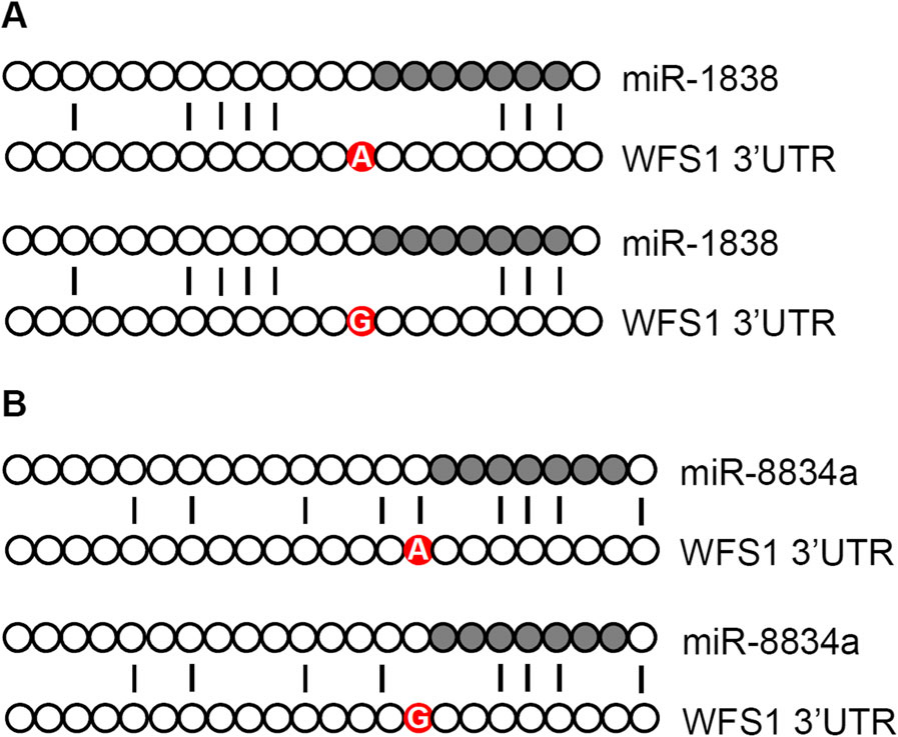
Sequence alignment of cfa-miR-1838 (***Panel* a**), and cfa-miR-8834a (***Panel* b**) microRNAs and their putative binding sites in the A and G allele of the *WFS1* gene

Sanger sequencing of the dog *WFS1* gene 3′ UTR region (1084 basepairs downstream the stop codon) verified the predicted rs852850348 A > G polymorphism in position 33 of the 3′ UTR as shown in Fig*.* 2. Additionally, Fig*.*2 demonstrates the sequence alignment of cfa-miR-1838 and cfa-miR-8834a and their binding sites in the *WFS1* mRNA with the “A” and “G” allele, respectively. It shows that the rs852850348 SNP is located 1 base pair next to the binding sites of the seed sequence of these microRNAs.

The seed sequence of miRNAs is shown in grey and the rs852850348 locus in red. Vertical lines represent complementary nucleotides between miRNA and mRNA.

### Genotyping

**Fig. 3:**
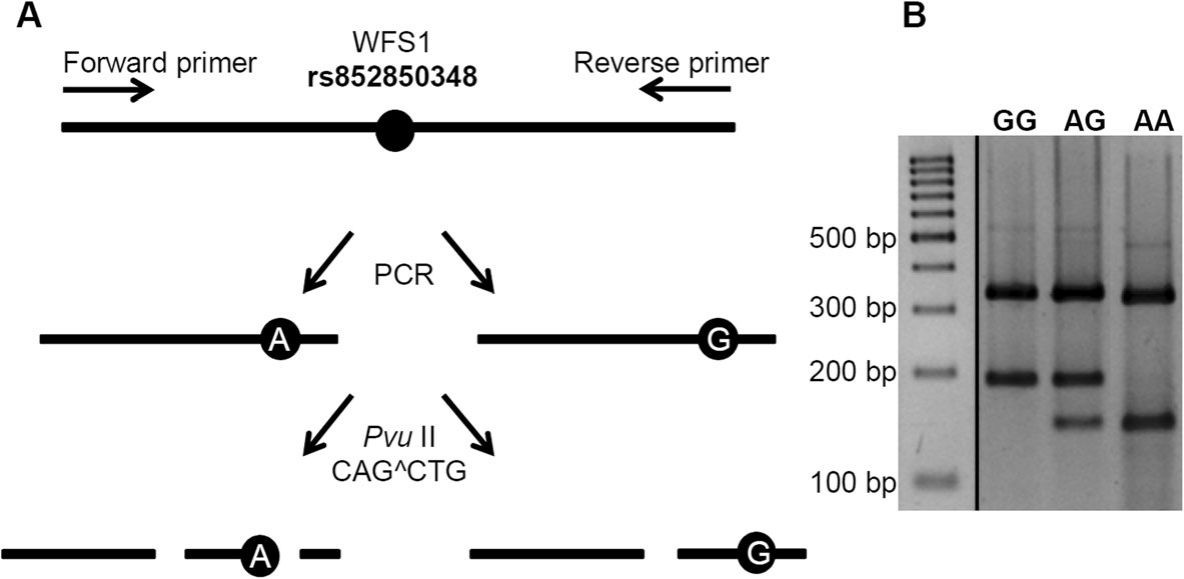
Genotyping of rs852850348 SNP by restriction digestion of PCR amplicons. ***Panel* a** shows the principle of the applied genotyping method. Amplicons of A allele were digested with the *Pvu*II enzyme into three fragments with different lengths, while in case of G allele only 2 fragments were generated. ***Panel* b** shows the separated fragments of various genotypes as detected by horizontal agarose gel electrophoresis

A genotyping method was designed for the analysis of rs852850348 using a PCR-RFLP technique. In case of “A” allele, the *Pvu*II enzyme digested the PCR product to three fragments with different lengths (334 + 148 + 46 bp), however, the sequence with “G” allele provided only two products (334 + 194 bp). The completion of restriction digestion were controlled by the 334 base pair-long fragment produced by a control cleavage site independent from the polymorphism (Fig*.* 3a). The obtained DNA fragments were detected by horizontal gel electrophoresis (Fig*.*3b).

**Fig. 4:**
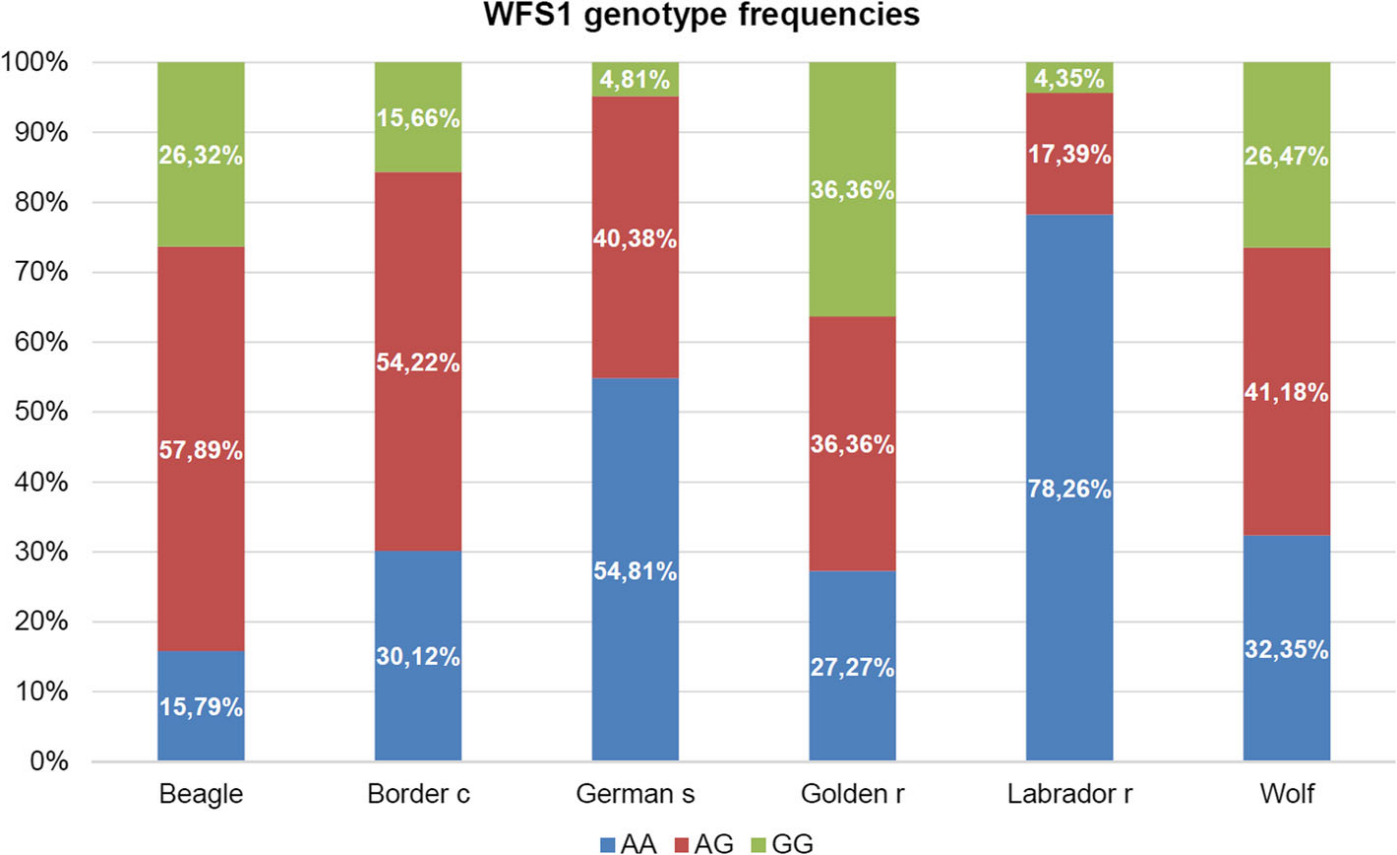
Variations in rs852850348 genotype distribution among the six subgroups

Using the above method, rs852850348 genotypes of 240 privately owned dogs from 5 different breeds and 34 wolves were determined, and the results are presented in Table 2 and Fig*.*3. In most breeds the G allele was the minor allele with a variable frequency from 13 to 47%, except for golden retrievers and beagles where its frequency rised up to 55% (Table 2). In accordance with the measured allele frequencies, genotype distribution showed also a variable picture (Fig*.* 4). The AA genotype was the most frequent among Labrador retrievers and less frequent among beagles.

**Table 2:** Allele frequencies of rs852850348 among the six subgroups

	Allele A	Allele G
**Border collie**	0.58	0.42
**German shepherd**	0.68	0.32
**Golden retriever**	0.45	0.55
**Labrador retriever**	0.87	0.13
**Beagle**	0.44	0.55
**Wolf**	0.53	0.47

### The impact of rs852850348 on luciferase signal

The molecular impact of rs852850348 SNP was analyzed by in vitro miRNA assay. As predicted by the miRBase database, two miRNAs were identified having a possible role in altering the gene expression. The entire 3’UTR region (524 base pairs) of the *WFS1* gene with the rs852850348 “A” allele variant was subcloned into a pMIR-Report luciferase reporter vector. Subsequently, the construct with the “G” allele at the rs852850348 locus was created by site directed mutagenesis. Moreover, a construct without any binding site of cfa-miR-8834a and cfa-miR-1838 (referred to as “control”) was also prepared.

**Fig. 5:**
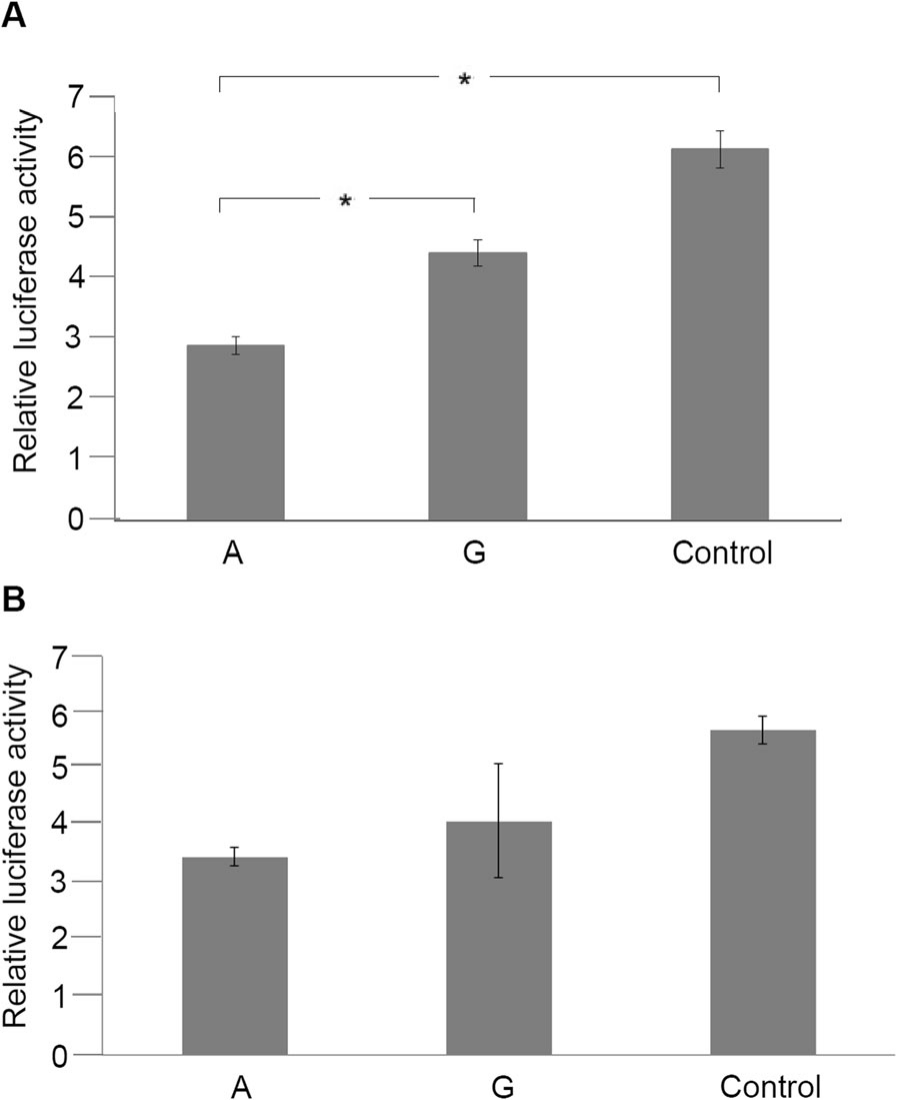
Luciferase reporter assay of *WFS1* 3’UTR allelic variants with co-infected cfa-miR-8834a (***Panel* a**) or cfa-miR-1838 (***Panel* b**) microRNA

Cotransfection of miR-8834a and the reporter construct containing the “A” allele of the rs852850348 SNP showed the lowest level of relative luciferase activity (47.15% of the “Control”). When miR-8834a was coinfected with the construct carrying “G” allele, 24,85% of increase was observed in relative luciferase activity comparing to the construct with “A” allele (72% of the control). Although the rs852850348 polymorphism is not located in the seed sequence of the miRNAs (see Fig*.*1), it is only one base pair adjacent to the seed sequence in both cases. Nonetheless, significant changes (*p* = 0.029) in luciferase activity were detected between the construct with an “A” and a “G” allele in the presence of miR-8834a, demonstrating that the mRNA-miRNA interaction was weaker in the presence of “G” allele. In addition, the “control” construct without a binding site of cfa-miR-8834a and cfa-miR-1838 resulted in the highest luciferase activity in the presence of cfa-miR-8834a. Additionally, significant changes were found between the construct containing “A” allele and the control (*p* = 0.022) (Fig*.* 5a).

Application of cfa-miR-1838 did not result in a significant decrease (*p* = 0.074) in relative luciferase level, and no significant differences were found between the relative luciferase activity of the allelic variants (Fig*.*5b).

**Fig. 6:**
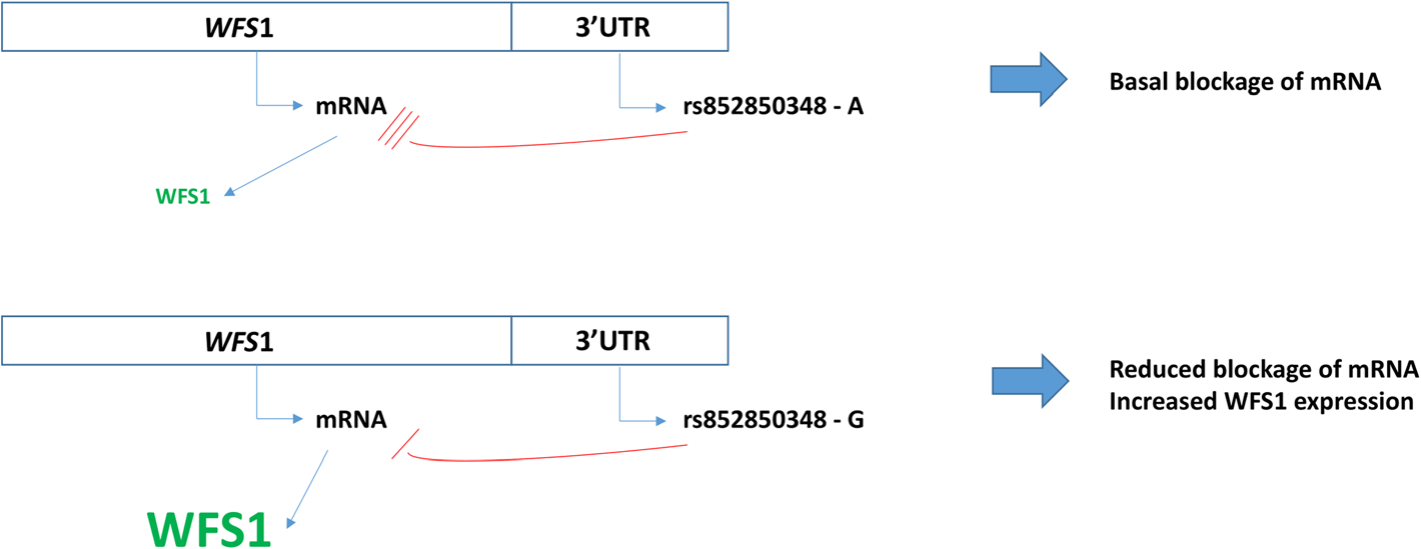
The possible functional role of the *WFS1* rs852850348 SNP

Figure 6 shows the potential functional consequence of this polymorphism when miR8834a binds to the 3’UTR of the dog *WFS1* gene.

The entire 3′ UTR region of the canine *WFS1* gene was subcloned into the pMIR luciferase riporter vector. Transient transfection were performed in HEK293 cells (for details, see Methods section). Luciferase activity values normalized to *β*-galactosidase activity were measured, Panel A represents average ± SD values of three independent experiments in case of co-transfection with cfa-miR-8834a. (* *p* < 0.05). The “A” luciferase construct harbored the rs852850348 A allele, whereas the “G” contained the rs852850348 G variant. “Control” construct had a different DNA-insert with the same length lacking any sequence complementary to cfa-miR-8834a. Panel B demonstrates average ± SD values of three independent experiments in case of cfa-miR-1838 co-transfection. The circumstances are the same as described before.

### Gene-behaviour association study

Both the inter-rater reliability and the test-retest reliability were satisfactory (ICC = 0.704 and 0.872, respectively, *N* = 14).

**Fig. 7:**
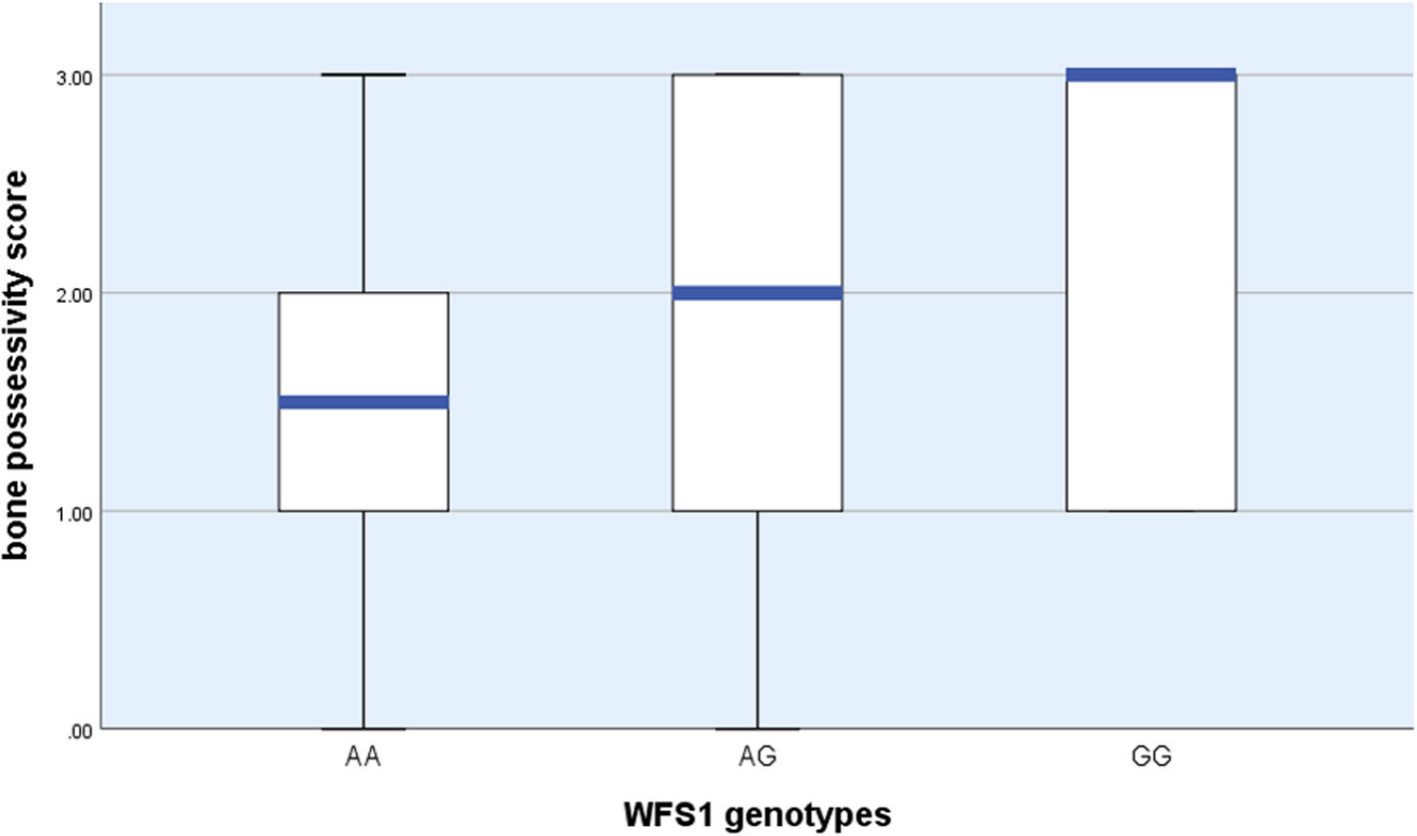
*WFS1* gene-behaviour associations in border collie dogs

The Generalized Linear Model has not revealed associations between the ‘Bone possessivity score’ and *WFS1* genotypes in the German shepherd dogs. However, in border collies, homozygote GG individuals were less inclined to leave the bone (i.e. they were more possessive) than homozygote AA dogs (Wald Chi square = 6.379, df = 2, *p* = 0.041, Fig*.* 7). Sex and age had no significant effect on the behaviour. Bone possessivity was not linked to aggressivity, as only 4 dogs growled and 2 attempted to bite during the test. The detailed data of the gene-behavior association study are available in the Supplementary material.

## Discussion

Very few canine polymorphisms can be found in databases compared to human polymorphism data. These few polymorphisms in databases are usually predicted by in silico methods. For this reason, first a bibliographic review was carried out for SNP candidates with high allele frequency and having a putative miRNA binding site. Previous studies also follow this study design when analyising dog polymorphisms being miRNA binding sites [29]. It is of importance to confirm the existence of in silico predicted SNPs in databases to be able to create a comprehensive database for dogs similar to other organisms.

It has recently been demonstrated that miRNAs play a crucial role in the regulation of protein synthesis, and polymorphisms located in the 3’UTR might alter the binding efficiency of miRNAs contributing to the fine tuning of this regulation. Several miRNA studies have been performed in the last couple of years in humans, mostly in clinical subjects [30–32] but there are some available data with behavioral aspects [33–35] as well. Behavioral traits and their connection with miRNAs were also investigated in our laboratory previously [16, 36, 37].

Although some miRNAs were proven to have a binding site in the 3’UTR of different canine genes [22], the functional effect of these miRNAs are still mostly unidentified, and data are available practically only in clinical settings [38–40]. The miRBase database (http://mirbase.org/) provides the sequence of 502 dog miRNAs, which are annotated in the dog genome (ftp://mirbase.org/pub/mirbase/CURRENT/genomes/cfa.gff3). A publication of an improved canine genome build, canFam3.1 [41] offers a chance to improve this knowledge: previously annotated dog miRNAs were validated and new miRNA sites were identified based on canFam3.1 genome assembly and RNA sequencing data [42]. According to our best knowledge, we are the first to report the functional effect of a SNP influencing the efficiency of microRNA binding in canine *WFS1* gene associated with behavioral traits.

The 3’UTR of the *WFS1* gene containing the rs852850348 SNP was investigated by in vitro luciferase reporter system. We observed that the expression of the reporter protein was significantly lower in the presence of the “A” allele compared to the “G” allele at the rs852850348 locus. This finding was of especial interest as the studied SNP was not located in the predicted seed sequence of the cfa-miR-8834a miRNA. This result confirms that although nucleotides at position 2 to 8 are crucial in miRNA–mRNA interaction, further bases of the mature miRNA can also influence this effect [43].

Please note, that the rs852850348 SNP site flanked the seed region. “A” allele is the major allele of rs852850348 SNP (http://www.ensembl.org/). Therefore, carrying the mutated alelle (“G”) does not result in high mRNA-miRNA interaction. Our results confirm the hypothesis that miRNAs usually negatively regulate protein expression in case of carrying wild type genotype [44]. However, the construct containing “G” allele showed also reduced luciferase activity compared to the control. This result suggests that one base pair change does not cause complete mRNA-miRNA dissociation. A similar finding was published by our laboratory previously [45]. The rs1046322 SNP in the 3’UTR of the human *WFS1* gene was also examined as putative miRNA binding site polymorphism. Its functional effect was demonstrated in a luciferase reporter system: the minor “A” allele showed lower repression compared to the major “G” allele, if co-expressed with miR-668 [16], similar to our results. Nothwithstanding, the rs852850348 SNP might be in the seed region of another, unknown miRNA. Therefore, the underpining molecular mechanism needs further studies.

Although in silico sequence alignment suggested the putative role of cfa-miR-1838 as well, it could not be confirmed by our luciferase reporter system. Neither significant decrease when applying this miRNA, nor any difference between the effect of the two allelic variants could be detected. These results suggest that this miRNA does not have a role in regulating *WFS1* expression.

Our association study suggests that the food possessivity behaviour might have genetic background in some breeds. Border collie dogs carrying GG genotype were more food possessive than dogs with AA genotypes. Food possessivity is often linked to possessive aggression, involving growling, baring the teeth, snapping, or biting when the dog possesses an object (food, bone, toy) and someone (family, stranger, animal) approaches and/or attempts to take it away [46, 47]. However, food possessivity was unrelated to aggression in our test, as only a few dogs growled or attempted to bite. The lack of association of bone possessivity to possessive agression in our study could be due to the low sample size, the unfamiliarity of the situation for the dogs, or the specific characteristics of the Border collie breed.

Wolframin is an endoplasmic reticulum membrane protein. Its function is not completely understood yet, however, its malfunction induces endoplasmic reticulum stress [18]. Endoplasmic reticulum stress activates the unfolded protein response and mediates the pathogenesis of psychiatric diseases when the damage occurs in the hippocampus, the amygdala and striatum [48], where wolframin is greatly present [17]. Therefore, food possession in dogs could be partially due to the polymorphism of the *WFS1* gene.

## Conlusions

In conclusion, our study suggests that cfa-miR-8834a regulates the dog *WFS1* protein expression, especially when carrying the major “A” allele of rs852850348 SNP located in the 3’UTR. Due to the observed biological effect, this locus is not a genetic marker only, but a functional polymorphism, suggesting that the modulation of *WFS1* expression might be an important factor in the molecular genetics of dog behavior. Additionally, this SNP might contribute to the genetic background of food possessivity behavior in dogs.

### Limitations of the study

The participating dogs vary in number and include 5 different breeds. Dogs are genetically diverse despite of being the same species. However, even though the breeds are not entirely homogeneous, only a small number of genes account for different phenotypes. Therefore, distinct dog breeds can be analysed together, however, the results have to be considered with caution.

## Supplementary information

**Additional file 1.**

## Data Availability

The dataset supporting the conclusions of this article is included within the article (and its additional file). The datasets generated and/or analysed during the current study are available in the GenBank (NC_006595.3), the Ensembl (ENSCAFG00000001781), the miRbase (MI0027920, MI0008044) and the dogSD (cfa6804527) repositories.

## Abbreviations

3’UTR: 3′ untranslated region
DMEM: Dulbecco’s Modified Eagle Medium
*DRD4*: Dopamine D4 receptor gene
HEK293T: Human Embryonic Kidney cell line
HWE: Hardy-Weinberg equilibrium
miRNA: microRNA
mRNA: Messenger RNA
*OPRM1*: Mu (μ) opioid receptor
*OXTR*: Oxytocin receptor gene
PBS: Phosphate-Buffered Saline
PCR: Polymerase chain reaction
SNP: Single nucleotide polymorphism
*TH*: Tyrosine hydroxylase gene
*WFS1*: Wolfram syndrome 1 gene
